# Paroxysmal Speech Arrest With Resolving Splenial and Symmetric White Matter Lesions in a Child: A Case Report

**DOI:** 10.7759/cureus.113992

**Published:** 2026-08-05

**Authors:** Panhui Yu, Fang Chen

**Affiliations:** 1 Department of Pediatric Neurology, Children's Hospital of Hebei Province, Shijiazhuang, CHN

**Keywords:** cytotoxic lesion of the corpus callosum, gjb1 differential diagnosis, mers type ii, paroxysmal speech arrest, pediatric neuroimaging, reversible splenial lesion syndrome, serial electroencephalography

## Abstract

Reversible splenial lesion syndrome (RESLES), mild encephalitis/encephalopathy with a reversible splenial lesion (MERS), and cytotoxic lesions of the corpus callosum (CLOCCs) are overlapping clinicoradiological constructs associated with infection, seizures, metabolic disturbance, and drug exposure. We report an 11-year-old right-handed boy with recurrent paroxysmal speech arrest over approximately 24 hours. During the episodes, consciousness and comprehension were preserved; he could vocalize and communicate by typing but could not produce meaningful speech. Transient left upper-limb or generalized weakness and upper respiratory symptoms accompanied the spells. Respiratory adenovirus testing was positive, and a *Mycoplasma pneumoniae *(*M. pneumoniae*)* *antibody titer was reported as 1:160, although respiratory and cerebrospinal fluid *M. pneumoniae* polymerase chain reaction testing was negative. Cerebrospinal fluid contained 1 white cell/microL, with normal protein, glucose, and chloride and mildly elevated interleukin-6 (IL-6). Baseline electroencephalography showed abundant right frontopolar, frontal, anterior temporal, and Fz interictal discharges without a captured electroclinical seizure. Magnetic resonance imaging demonstrated a splenial lesion with symmetric posterior periventricular and centrum semiovale abnormalities, compatible with a type II MERS/RESLES pattern or CLOCC-spectrum process. He received two 40 g doses of intravenous immunoglobulin (IVIG) and later intravenous methylprednisolone followed by oral prednisone, together with empirical acyclovir, azithromycin, mannitol, and supportive care. No further speech arrest or weakness was documented on or after illness day 5, and methylprednisolone was initiated on illness day 6. Follow-up electroencephalography on illness day 48 showed qualitatively fewer discharges confined to sleep, but no habitual event. Serial imaging showed lesion regression with residual fluid-attenuated inversion recovery (FLAIR) signal on illness day 74. At approximately 4.5 months, he remained asymptomatic during prednisone tapering. Because formal language and motor-speech testing was not performed during an episode, aphasia could not be definitively distinguished from anarthria, dysarthria, apraxia of speech, or focal speech arrest. This case highlights the diagnostic overlap among resolving callosal-white matter lesions, transient speech-output disturbance, and interictal epileptiform abnormalities, while cautioning against attributing recovery to a single treatment or equating residual magnetic resonance signal with active inflammation.

## Introduction

Mild encephalitis/encephalopathy with a reversible splenial lesion (MERS) was initially described as a clinicoradiological syndrome with transient neurological symptoms and a lesion in the splenium of the corpus callosum [1]. Reversible splenial lesion syndrome (RESLES) was subsequently proposed to encompass a wider range of triggers and clinical contexts [2]. The term cytotoxic lesions of the corpus callosum (CLOCCs) is a broader radiological descriptor because similar lesions may extend beyond the splenium, may not be accompanied by mild encephalopathy, and may not completely disappear on early follow-up imaging [3,4].

In children, MERS/RESLES is frequently reported in association with infection and generally has a favorable outcome [5,6]. Type I MERS consists of an isolated lesion in the splenium of the corpus callosum, whereas type II MERS includes additional symmetric abnormalities in the adjacent callosal fibers or bilateral cerebral white matter. When abnormalities are symmetric and extend beyond the splenium, the differential diagnosis includes infectious or immune-mediated encephalitis, inflammatory demyelination, seizure-related changes, and metabolic or genetic white matter disorders. We describe a child in whom recurrent paroxysmal speech arrest was the unusual dominant presentation, accompanied by longitudinal interictal electroencephalographic abnormalities and resolving splenial and bilateral white matter lesions. This case highlights the value of combining careful bedside characterization with serial electroencephalography and magnetic resonance imaging (MRI) while maintaining a broad differential diagnosis.

## Case presentation

Clinical presentation

Illness day 1 was defined as the day the first prolonged speech-arrest episode began. A right-handed 11-year-old boy was admitted on illness day 2 after several recurrent episodes of inability to produce meaningful speech within approximately 24 hours. During the first prolonged episode, he could vocalize but could not form words; he also described tongue stiffness and slurred speech. Consciousness and comprehension were preserved, and he communicated by typing on a mobile phone. The first episode lasted approximately four hours, and later recurrences resolved within about one hour. The spells were accompanied by transient left upper-limb discomfort or weakness and, during the final recurrence, generalized weakness. There was no documented loss of consciousness, convulsive activity, or urinary or fecal incontinence. Nasal obstruction, throat discomfort, and intermittent cough were also present. A similar transient episode had reportedly occurred at five years of age, but the original records and imaging were unavailable.

On admission, temperature was 36.4°C, heart rate was 108 beats/min, respiratory rate was 22 breaths/min, and blood pressure was 122/72 mmHg. He was alert, followed commands, and had no meningeal signs. Cranial nerve examination was unremarkable. Left upper-limb strength was initially graded 4/5; strength and tone in the remaining limbs were normal, deep tendon reflexes were symmetric, and pathological reflexes were absent. Brief recurrences of the speech disturbance and weakness occurred early in the admission, without fever, convulsion, or sustained impairment of consciousness. The presentation prompted consideration of focal seizures, infection-associated encephalopathy, inflammatory or demyelinating disease, and metabolic or genetic white matter disorders.

Diagnostic evaluation

Routine hematologic, biochemical, metabolic, thyroid, coagulation, and cardiac enzyme screening did not identify an explanatory abnormality, and cranial computed tomography was unremarkable. Respiratory adenovirus nucleic acid testing was positive. The laboratory report listed a *Mycoplasma pneumoniae* (*M. pneumoniae*) antibody titer of 1:160; the assay class and institutional positivity threshold were not available in the extracted record, and paired serology was not obtained. Respiratory* M. pneumoniae* DNA testing was negative. Chest computed tomography showed inflammatory changes in the left lower lobe.

Lumbar puncture was performed on illness day 4 before intravenous immunoglobulin (IVIG) or glucocorticoid treatment. Opening pressure was 200 mmH_2_O, within a published pediatric reference interval of 115-280 mmH2O [7]. Cerebrospinal fluid (CSF) was colorless and clear, with 1 white cell/microL (reference range: 0-10 cells/microL), total protein of 0.15 g/L (reference range: 0.08-0.43 g/L), glucose of 3.75 mmol/L (reference range: 2.5-4.4 mmol/L), chloride of 123.1 mmol/L (reference range: 120-132 mmol/L), and adenosine deaminase of <4 U/L (reference range: 0-8 U/L). The CSF red-cell count was 200 cells/microL; the institutional report did not provide a reference interval for this parameter. Key CSF findings are summarized in Table 1.

**Table 1:** Key cerebrospinal fluid routine and biochemical findings *The opening-pressure interval is from a prospective pediatric reference study [7]. Other intervals are those printed on the institutional laboratory reports. One cell/microL is equivalent to 1 x 10^6^/L

Parameter	Patient value	Reference range	Interpretation
Opening pressure	200 mmH2O	115-280 mmH2O*	Within published pediatric interval
Appearance	Colorless; clear	Colorless; clear	Normal
Protein qualitative test	Negative	Negative	Normal
White cells	1 cell/microL	0-10 cells/microL	No pleocytosis
Red cells	200 cells/microL	Not provided by laboratory	Reported without an institutional interval
Mononuclear fraction	100%	Not provided by laboratory	Based on a total of one white cell
Total protein	0.15 g/L	0.08-0.43 g/L	Within range
Glucose	3.75 mmol/L	2.5-4.4 mmol/L	Within range
Chloride	123.1 mmol/L	120-132 mmol/L	Within range
Adenosine deaminase	<4 U/L	0-8 U/L	Within range

CSF bacterial culture, India ink examination, and mycobacterial staining were negative. Polymerase chain reaction testing of CSF for *M. pneumoniae*, herpes simplex virus types 1 and 2, Epstein-Barr virus, cytomegalovirus, and enteroviruses was negative. CSF interleukin-6 was 11.22 pg/mL (reference: <6.00 pg/mL); the other 13 measured cytokines were within their respective laboratory reference intervals. Comprehensive neural autoantibody testing for autoimmune encephalitis or demyelinating disease and expanded genetic or metabolic testing were recommended but were not completed after discussion with the family.

The initial four-hour video electroencephalogram (EEG), performed on illness day 3, showed a mildly slow background. During wakefulness and sleep, abundant sharp waves, sharply contoured slow waves, and brief fast rhythmic activity were recorded over the right frontopolar, frontal, anterior temporal, and midline Fz regions. The abnormalities occurred sporadically, in runs, and at times continuously; occasional left-sided discharges were also reported. No habitual clinical event was captured, and the report did not describe ictal evolution.

Brain MRI on illness day 4 demonstrated symmetric abnormalities involving the splenium of the corpus callosum, bilateral posterior periventricular white matter, and bilateral centrum semiovale. The distribution was broader than an isolated splenial lesion and was interpreted as compatible with a type II MERS/RESLES pattern or CLOCC-spectrum process. Magnetic resonance angiography and venography showed no vascular abnormality. Whole-spine MRI was normal, and orbital MRI showed no optic nerve abnormality; mild paranasal sinus mucosal thickening was noted.

Treatment and inpatient course

The patient received IVIG 40 g on illness days 4 and 5 (total 80 g), without a documented infusion reaction. Intravenous methylprednisolone sodium succinate was administered at 800 mg once daily on illness days 6-8, 400 mg once daily on illness days 9-11, 200 mg once daily on illness days 12-14, and 100 mg once daily on illness days 15-17. Intravenous methylprednisolone was discontinued on illness day 18 and replaced with oral prednisone 55 mg/day.

Empirical acyclovir was continued through illness day 14 while viral encephalitis was being excluded. A five-day course of azithromycin was administered for possible *M. pneumoniae*-associated respiratory infection. Mannitol was used early in the admission, then reduced and discontinued by illness day 9; supportive and neuroprotective treatment was also provided. No antiseizure medication was documented. No electroclinical seizure was captured, and the spells did not recur after illness day 5; these features favored continued clinical observation.

No further speech arrest was documented on or after illness day 5, and limb strength had returned to normal before methylprednisolone was initiated on illness day 6. The patient remained free of seizures, sustained altered consciousness, and recurrent focal weakness. He was discharged clinically stable on illness day 19 while taking oral prednisone 55 mg/day.

Serial imaging, electroencephalography, and follow-up

**Figure 1:**
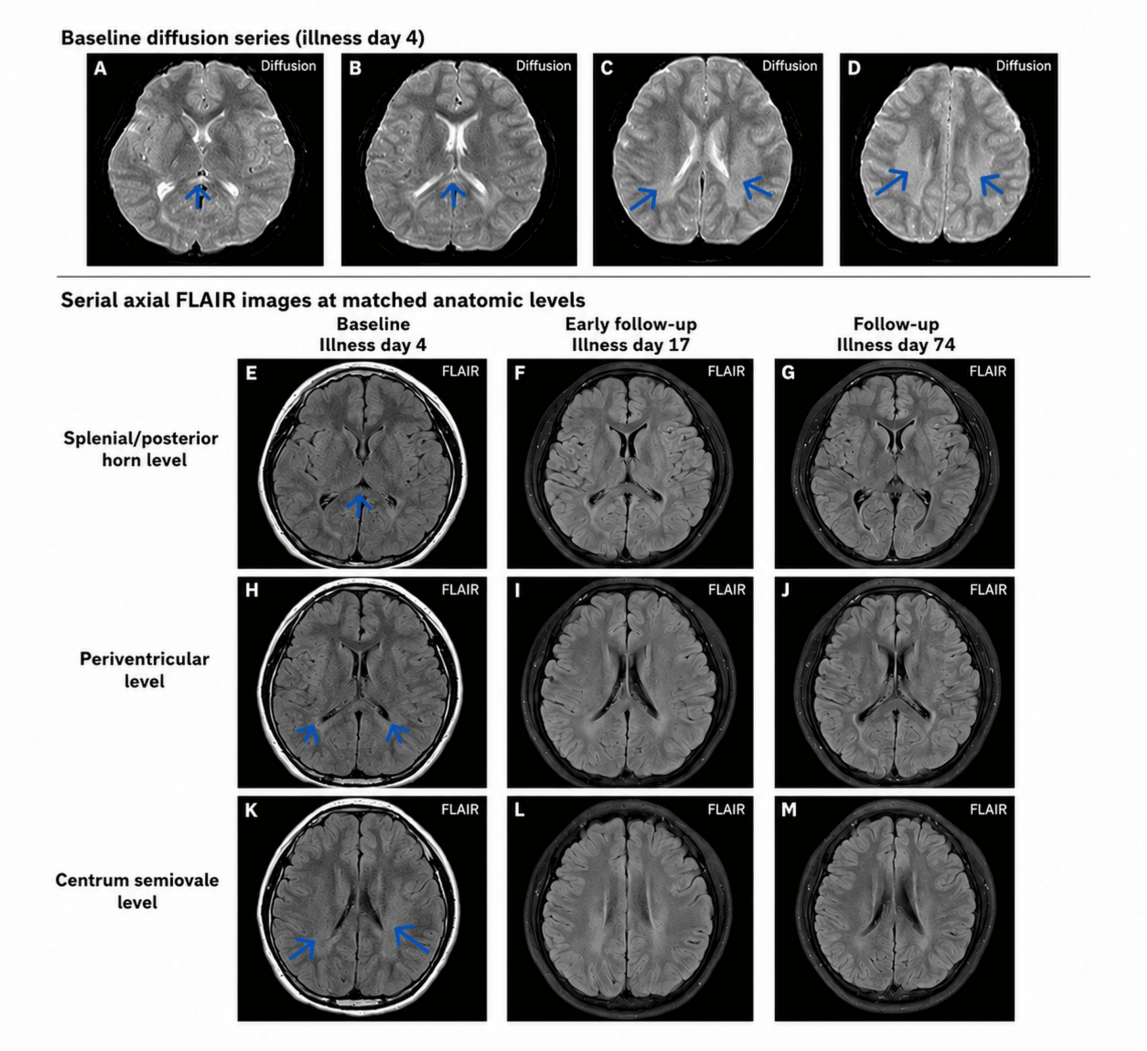
Serial brain MRI evolution FLAIR: fluid-attenuated inversion recovery; MRI: magnetic resonance imaging (A-D) Baseline diffusion-series images obtained on illness day 4 illustrate the distribution of the acute abnormalities. Blue arrows indicate representative splenial, bilateral posterior periventricular, and centrum semiovale abnormalities; paired apparent diffusion coefficient maps were not available in the exported material. (E-M) Matched axial fluid-attenuated inversion recovery images at the splenial/posterior horn level (E-G), periventricular level (H-J), and centrum semiovale level (K-M) demonstrate reduced lesion extent on the early follow-up scan obtained on illness day 17 and persistent but substantially regressed abnormalities on illness day 74. The corresponding baseline FLAIR abnormalities are highlighted by blue arrows in panels E, H, and K. No new or enlarging lesion was reported. Panels were cropped and assembled from de-identified clinical images; signal intensity, contrast, and the underlying image data were not altered

An early follow-up MRI on illness day 17 showed reduced lesion extent. MRI on illness day 46 continued to show residual abnormalities. On illness day 74, the splenial and bilateral white matter lesions remained substantially smaller than at baseline, with no new or enlarging lesion, but there was no appreciable interval change compared with illness day 46 (Figure 1). Complete radiological resolution had therefore not been documented.

**Figure 2:**
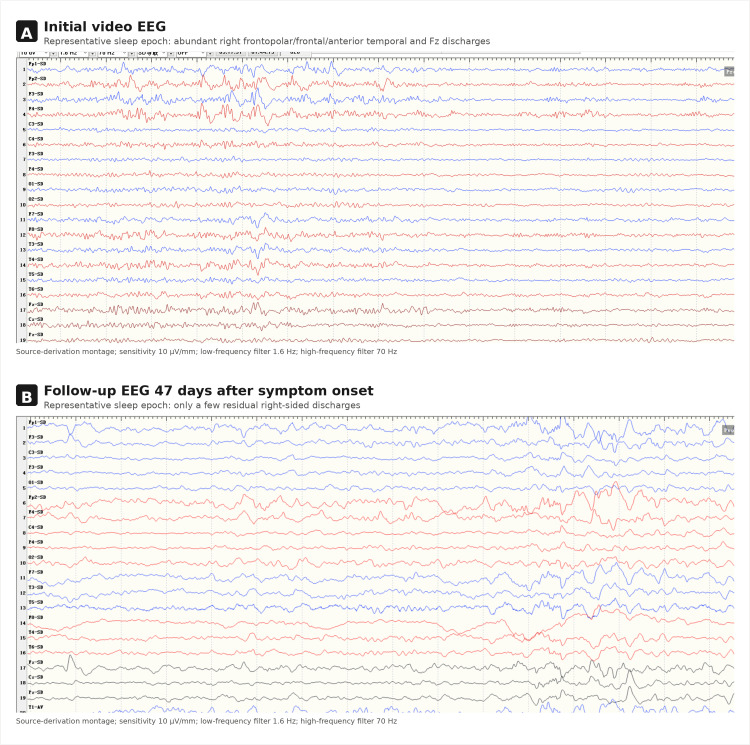
Serial interictal electroencephalographic findings EEG: electroencephalogram (A) The initial four-hour video EEG performed on illness day 3 showed a mildly slow background and abundant sharp waves, sharply contoured slow waves, and brief fast rhythmic activity over the right frontopolar, frontal, anterior temporal, and midline Fz regions during wakefulness and sleep. The abnormalities occurred sporadically, in runs, and at times continuously; occasional left-sided discharges were also reported. (B) Repeat EEG performed on illness day 48, approximately 47 days after symptom onset, showed persistent mild background slowing but a qualitatively reduced interictal burden, with only a few right frontopolar, frontal, anterior temporal, and Fz discharges confined to sleep and occurring sporadically or in brief runs. No habitual clinical event was captured during either recording, and neither report described ictal evolution. Representative sleep epochs are displayed using a source-derivation montage with a sensitivity of 10 microV/mm, a low-frequency filter of 1.6 Hz, and a high-frequency filter of 70 Hz. Panels were cropped and assembled from de-identified EEG screenshots; the underlying signal traces were not altered

Repeat EEG on illness day 48 was performed while the child remained clinically asymptomatic and was receiving oral prednisone. The recording lasted 109 minutes and included 33 minutes of sleep. Background activity remained mildly slow, but only a few sharp waves, sharply contoured slow waves, and brief fast rhythmic abnormalities were seen over the right frontopolar, frontal, anterior temporal, and Fz regions. These abnormalities were confined to sleep and occurred sporadically or in brief runs. No habitual clinical event was captured, and the report did not describe ictal evolution. Compared with the initial four-hour study, the interictal abnormality had qualitatively decreased (Figure 2); the unequal recording durations and sleep sampling precluded a standardized quantitative comparison.

Oral prednisone remained at 55 mg/day until illness day 75, when a taper of 5 mg every seven days was begun. A dose of 15 mg/day was documented on illness day 123. At follow-up on illness day 137, approximately 4.5 months after onset, the patient had no recurrent speech disturbance, seizure, behavioral change, or limb dysfunction, and neurological examination was normal. The plan was to reduce prednisone to 10 mg/day for two weeks and then 5 mg/day for two weeks, followed by repeat MRI. No outcome after complete glucocorticoid withdrawal was available. The major clinical, treatment, neurophysiological, and imaging milestones are summarized in Table 2.

**Table 2:** Clinical, treatment, imaging, and EEG timeline EEG: electroencephalogram; MRI: magnetic resonance imaging; IVIG: intravenous immunoglobulin; CSF: cerebrospinal fluid;

Illness day/time point	Clinical or diagnostic finding	Treatment or outcome
Age 5 years	A similar transient speech-output episode was reported; original records and imaging were unavailable	No verifiable treatment data
1-2	Recurrent speech arrest with preserved consciousness, comprehension, and typing; transient left upper-limb or generalized weakness; upper respiratory symptoms	Admitted on day 2
3	Initial four-hour video EEG: mildly slow background; abundant right frontopolar/frontal/anterior temporal/Fz interictal abnormalities; no habitual event or ictal evolution	No antiseizure medication documented
4	MRI: splenial, posterior periventricular, and centrum semiovale abnormalities. CSF obtained before immunotherapy: no pleocytosis or biochemical abnormality; IL-6 mildly elevated	IVIG 40 g; empirical anti-infective and supportive therapy
5	No further speech arrest documented; strength returned to normal	Second IVIG 40 g
6-8	Clinically stable without recurrent focal deficit	Methylprednisolone 800 mg IV once daily
9-11	No recurrent speech arrest or seizure	Methylprednisolone 400 mg IV once daily; mannitol discontinued by day 9
12-14	Continued clinical stability	Methylprednisolone 200 mg IV once daily; acyclovir completed on day 14
15-17	Early follow-up MRI on day 17 showed reduced lesion extent	Methylprednisolone 100 mg IV once daily
18-19	Clinically stable without speech arrest, seizure, or focal deficit	Changed to oral prednisone 55 mg/day on day 18; discharged on day 19
46	Residual MRI abnormalities persisted	Outpatient follow-up
48	Repeat EEG: persistent mild background slowing; only a few right-sided discharges confined to sleep; no habitual event	Continued oral prednisone.
74-75	MRI on day 74: lesions substantially smaller than baseline and stable compared with day 46; no new lesions	Prednisone taper began on day 75, decreasing 5 mg every seven days
123	No recurrent neurological symptoms documented	Prednisone 15 mg/day documented
137	Asymptomatic; neurological examination normal	Plan: prednisone 10 mg/day for two weeks, then 5 mg/day for two weeks; repeat MRI planned

## Discussion

The imaging pattern in this patient is best described as a resolving type II MERS/RESLES pattern within the CLOCC spectrum. Involvement of the splenium together with symmetric posterior periventricular and centrum semiovale abnormalities was broader than classic type I MERS. Because the child did not have sustained encephalopathy and complete radiological disappearance had not been demonstrated, the descriptive terms "resolving" and "CLOCC-spectrum" are preferable to assuming complete reversibility or a single pathophysiological mechanism.

The respiratory findings provide a possible trigger rather than proof of a specific causal infection. *M. pneumoniae*-associated MERS has been described in children [8], and adenovirus-associated type II MERS has also been reported [9]. In this case, however, *M. pneumoniae* respiratory and CSF polymerase chain reaction tests were negative; only a single antibody titer was available, adenovirus was detected in the respiratory tract rather than the CSF, and routine CSF findings were noninflammatory. The safest interpretation is therefore that the neurological syndrome was temporally associated with a respiratory infection and may have been infection-triggered.

The term speech arrest was chosen deliberately. Preserved consciousness, comprehension, and the ability to type suggested that conceptual language formulation was at least partly preserved, whereas inability to produce spoken words, tongue stiffness, and slurring raised a motor-speech or speech-output disorder. Because formal language and motor-speech testing was not performed during an episode, aphasia could not be definitively distinguished from anarthria, dysarthria, apraxia of speech, or focal speech arrest. A closely overlapping phenotype of sudden aphasia or speech disturbance with unilateral weakness and callosal-bilateral white matter lesions has been reported in pediatric siblings, including recurrence in one child [10].

The EEG findings complicated attribution of the spells to the MRI lesions alone. Abundant right frontopolar, frontal, anterior temporal, and Fz interictal abnormalities were present initially and qualitatively decreased on the follow-up study performed on illness day 48. This parallel improvement is compatible with a transient network disturbance during the encephalopathic process, but it does not establish an epileptic mechanism for the original speech events. Neither recording captured a habitual event or ictal evolution; the recordings differed in duration and sleep sampling, and language dominance was not tested. The right-sided discharges in a right-handed child, therefore, cannot be taken as proof of focal epileptic aphasia or postictal dysfunction. In this context, continued observation without antiseizure medication was considered reasonable, although an epileptic mechanism could not be fully excluded.

An inherited disorder deserves specific consideration. GJB1-related X-linked Charcot-Marie-Tooth disease type 1 (CMTX1) can cause acute, self-limited episodes of dysarthria, lingual or limb weakness, and symmetric reversible white matter abnormalities, often in boys and sometimes during physiological stress or infection [11]. The male sex, reported episode at age five years, recurrent speech and weakness symptoms, and symmetric callosal-deep white matter distribution overlap with this phenotype. No peripheral neuropathy phenotype was documented, but formal nerve-conduction studies and GJB1 testing were not performed. Recurrence should prompt detailed maternal-family history, examination for distal weakness or foot deformity, nerve-conduction testing, and targeted genetic evaluation, in addition to reassessment for autoimmune, demyelinating, and metabolic mimics.

Delayed radiological resolution is also clinically relevant. Splenial and adjacent white matter abnormalities may follow different time courses, and residual fluid-attenuated inversion recovery (FLAIR)or T2 signal can persist after diffusion abnormalities and neurological symptoms improve [12,13]. In this patient, the illness-day-74 scan remained abnormal but showed substantial regression from baseline and no interval progression compared with illness day 46. Persistent but regressing signal, in the absence of clinical relapse or new lesions, did not by itself demonstrate ongoing active inflammation.

Treatment effects cannot be inferred from this single case. The patient received IVIG, glucocorticoids, acyclovir, azithromycin, mannitol, and supportive therapy in close temporal proximity, while MERS/RESLES frequently has a favorable natural course. Importantly, no further speech arrest or weakness was documented on or after illness day 5, before methylprednisolone began on illness day 6. Recovery therefore cannot be attributed to glucocorticoids, and the role of IVIG likewise cannot be separated from spontaneous improvement or the other treatments. The prolonged prednisone course reflected early concern for immune-mediated encephalitis or demyelination. Once clinical stability and non-progressive imaging were established, gradual reduction was reasonable because prolonged systemic glucocorticoid therapy in children can suppress the hypothalamic-pituitary-adrenal axis [14]. At the last follow-up, however, the patient had not yet completed prednisone withdrawal, so the safety or efficacy of the taper cannot be concluded.

This report has several limitations. The episode at age five years could not be verified. No formal language or motor-speech examination was performed during a spell, and no habitual spell was captured on EEG. The two EEG recordings were not matched in duration or sleep sampling, preventing quantitative comparison, and language dominance was not assessed. The causal role of adenovirus or *M. pneumoniae* remained presumptive. Comprehensive neural autoantibody, nerve-conduction, GJB1, and expanded genetic or metabolic testing were not completed. Multiple concurrent therapies and the favorable natural history of RESLES preclude attribution of treatment efficacy. Finally, follow-up was approximately 4.5 months; formal neuropsychological assessment was not performed, the child remained on prednisone, complete radiological resolution had not been documented, and no postwithdrawal clinical, EEG, or MRI outcome was available.

## Conclusions

Paroxysmal speech arrest can occur with resolving splenial and symmetric bilateral white matter lesions in a child, but preserved typing and comprehension do not by themselves distinguish aphasia from anarthria, dysarthria, apraxia of speech, or focal speech arrest. Serial EEG in this case showed a qualitative reduction in right frontal interictal abnormalities, yet the absence of a captured habitual event or ictal evolution prevented confirmation of an epileptic mechanism. Clinical recovery preceded methylprednisolone treatment, and residual but regressing FLAIR signal persisted after symptoms resolved; neither treatment efficacy nor active inflammation should be inferred from these observations alone. Recurrent episodes should prompt repeat EEG and MRI and renewed evaluation for epilepsy, immune-mediated or demyelinating disease, metabolic disorders, and GJB1-related CMTX1.

## References

[1] Tada H, Takanashi J, Barkovich AJ (2004). Clinically mild encephalitis/encephalopathy with a reversible splenial lesion. Neurology.

[2] Garcia-Monco JC, Cortina IE, Ferreira E, Martínez A, Ruiz L, Cabrera A, Beldarrain MG (2011). Reversible splenial lesion syndrome (RESLES): what's in a name?. J Neuroimaging.

[3] Starkey J, Kobayashi N, Numaguchi Y, Moritani T (2017). Cytotoxic lesions of the corpus callosum that show restricted diffusion: mechanisms, causes, and manifestations. Radiographics.

[4] Moors S, Nakhostin D, Ilchenko D, Kulcsar Z, Starkey J, Winklhofer S, Ineichen BV (2024). Cytotoxic lesions of the corpus callosum: a systematic review. Eur Radiol.

[5] Chen H, Yu X, Chen Y, Wu H, Wu Z, Zhong J, Tang Z (2023). Reversible splenial lesion syndrome in children: a retrospective study of 130 cases. Front Neurol.

[6] Kontzialis M, Soares BP, Huisman TA (2017). Lesions in the splenium of the corpus callosum on MRI in children: a review. J Neuroimaging.

[7] Avery RA, Shah SS, Licht DJ (2010). Reference range for cerebrospinal fluid opening pressure in children. N Engl J Med.

[8] Ueda N, Minami S, Akimoto M (2016). Mycoplasma pneumoniae-associated mild encephalitis/encephalopathy with a reversible splenial lesion: report of two pediatric cases and a comprehensive literature review. BMC Infect Dis.

[9] Akın ME, Çıtak-Kurt AN, Bayhan Gİ, Ezgü ZD, Bulut SŞ (2021). Mild encephalopathy with reversible extensive white matter lesions in a child with acute adenoviral infection and a literature review. Turk J Pediatr.

[10] Chen Q, Huang J, Huang J (2025). Cytotoxic lesions of the corpus callosum (CLOCC) in siblings: a case report. Curr Med Imaging.

[11] Tian D, Zhao Y, Zhu R, Li Q, Liu X (2021). Systematic review of CMTX1 patients with episodic neurological dysfunction. Ann Clin Transl Neurol.

[12] Ueda F, Yoshie Y, Aburano H, Hashimoto M, Matsui O, Gabata T (2014). Splenial and white matter lesions showing transiently-reduced diffusion in mild encephalopathy monitored with MR spectroscopy and imaging. Magn Reson Med Sci.

[13] Takanashi J, Imamura A, Hayakawa F, Terada H (2010). Differences in the time course of splenial and white matter lesions in clinically mild encephalitis/encephalopathy with a reversible splenial lesion (MERS). J Neurol Sci.

[14] Improda N, Chioma L, Capalbo D, Bizzarri C, Salerno M (2025). Glucocorticoid treatment and adrenal suppression in children: current view and open issues. J Endocrinol Invest.

